# Silencing of *Sphingosine kinase 1* Affects Maturation Pathways in Mouse Neonatal Cardiomyocytes

**DOI:** 10.3390/ijms22073616

**Published:** 2021-03-31

**Authors:** Ewelina Jozefczuk, Piotr Szczepaniak, Tomasz Jan Guzik, Mateusz Siedlinski

**Affiliations:** 1 Department of Internal and Agricultural Medicine, Faculty of Medicine, Jagiellonian University Medical College, 31-121 Cracow, Poland; ewelina.jozefczuk@doctoral.uj.edu.pl (E.J.); piotr.szczepaniak@uj.edu.pl (P.S.); t.guzik@uj.edu.pl (T.J.G.); 2 Institute of Cardiovascular and Medical Sciences, BHF Glasgow Cardiovascular Research Centre, University of Glasgow, Glasgow G12 8TA, UK

**Keywords:** sphingosine kinase-1, sphingosine-1-phosphate, cardiomyocyte, cardiomyocyte hypertrophy, cardiomyocyte maturation, cardiomyocyte proliferation

## Abstract

Sphingosine kinase-1 (Sphk1) and its product, sphingosine-1-phosphate (S1P) are important regulators of cardiac growth and function. Numerous studies have reported that Sphk1/S1P signaling is essential for embryonic cardiac development and promotes pathological cardiac hypertrophy in adulthood. However, no studies have addressed the role of Sphk1 in postnatal cardiomyocyte (CM) development so far. The present study aimed to assess the molecular mechanism(s) by which *Sphk1* silencing might influence CMs development and hypertrophy in vitro. Neonatal mouse CMs were transfected with siRNA against *Sphk1* or negative control, and subsequently treated with 1 µM angiotensin II (AngII) or a control buffer for 24 h. The results of RNASeq analysis revealed that diminished expression of *Sphk1* significantly accelerated neonatal CM maturation by inhibiting cell proliferation and inducing developmental pathways in the stress (AngII-induced) conditions. Importantly, similar effects were observed in the control conditions. Enhanced maturation of *Sphk1*-lacking CMs was further confirmed by the upregulation of the physiological hypertrophy-related signaling pathway involving Akt and downstream glycogen synthase kinase 3 beta (Gsk3β) downregulation. In summary, we demonstrated that the *Sphk1* silencing in neonatal mouse CMs facilitated their postnatal maturation in both physiological and stress conditions.

## 1. Introduction

Adult mammalian hearts lack significant self-regenerative capacity [1]. When subjected to different types of stress, both physiological and pathological, cardiomyocytes (CMs) respond through hypertrophic growth [2]. Physiological hypertrophy is a hallmark of postnatal maturation of CMs, whereas in adulthood, it may be induced by physical exercise or during pregnancy [3,4]. Pathological cardiac hypertrophy appears as a consequence of chronic pressure or volume overload. Although such adaptation is initially desired, since it helps to maintain the proper function of cardiac muscle, excessive remodeling is maladaptive and predisposes to heart failure. Despite the fact that both physiological and pathological hypertrophy result in cardiomyocyte growth in volume/size, these processes are mediated by divergent, not yet fully understood, molecular mechanisms [2].

Sphingosine kinase 1 (Sphk1) is an enzyme responsible for the production of sphingosine-1-phosphate (S1P), a lysophopholipid acting as an extracellular ligand for its cognate receptors (sphingosine-1-phosphate receptors, S1Prs) or as an intracellular signaling mediator, governing many cellular processes such as growth, proliferation, maturation and apoptosis. The significance of Sphk1/S1P/S1Prs signaling in cardiac biology is evident, as reported by numerous studies [5]. It has been recognized as an important regulator of embryonic vasculature and heart development [5]. Global *S1pr1*, *S1pr1/S1pr2/S1pr3* and *Sphk1/Sphk2* knockout mice are characterized by defective vasculogenesis and are embryonic lethal [6,7,8]. CM-specific *S1Pr1* knockout mice die perinatally due to cardiogenesis defects [9]. Moreover, cardiac endothelium-specific deletion of *S1pr1* results in abnormal coronary vascular formation and insufficient atrial and ventricular development, and such mice die in utero [10]. Although the crucial role of S1P signaling in embryonic cardiology is widely acknowledged, there are no reports concerning its function in the context of physiological hypertrophy associated with postnatal CM maturation.

In the adult heart, S1P signaling has been linked to detrimental pro-hypertrophic properties [11,12,13]. Our study revealed significant protection against angiotensin II-induced cardiac hypertrophy of *Sphk1* knockout mice as compared with wild-type mice [14]. Furthermore, this finding was supported by a pharmacological study using a selective inhibitor of Sphk1-PF543 [15]. Interestingly, while angiotensin II (AngII) is primarily known to induce pathological hypertrophy of CMs, upregulation of AngII expression also accompanied physiological hypertrophy, and increased angiotensin Type I receptor (Agtr1) signaling was necessary for the induction of physiological growth of neonatal CMs [16,17].

The present study investigated the in vitro effects of *Sphk1* silencing on neonatal mouse CM transcriptome, as well as key cardiac proteins in the presence of AngII and in control conditions.

## 2. Results

### 2.1. Sphk1 Silencing Affects the Neonatal CM Transcriptome in the Presence of AngII

**Figure 1:**
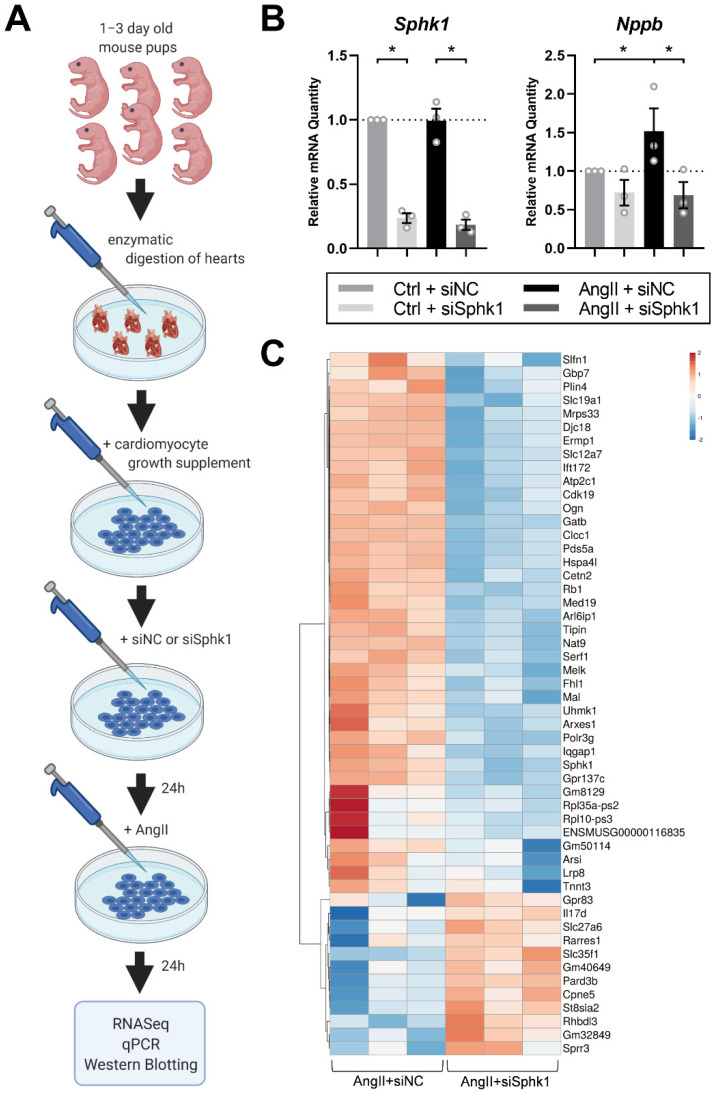
Sphingosine kinase 1 (*Sphk1*) silencing affects the transcriptome of angiotensin II (AngII)-treated neonatal cardiomyocytes (CMs). (**A**) Schematic representation of the experimental design (created with BioRender.com). (**B**) Expression of *sphingosine kinase-1* (*Sphk1*) and *natriuretic peptide type B* (*Nppb*) quantified using real-time polymerase chain reaction in neonatal CMs transfected with siNC or siSphk1 and treated with 1 µM AngII or a control buffer (Ctrl) for 24 h. Each point corresponds to an independent experiment (biological replicates, each performed in quadruplicate). Data are represented as means ± SEM, * *p* < 0.05; (**C**) All 52 genes for which mRNA expression was >2.5 times up- or downregulated (FDR *p* < 0.05) by siSphk1 treatment in neonatal CMs. The heatmap, created with Clustvis [19], shows the results from three independent sets of experiments.

To examine the effects of *Sphk1* silencing on hypertrophy development in neonatal CMs in vitro, CMs were transfected with siRNA against *Sphk1* (siSphk1) or negative control siRNA (siNC), and treated with AngII or a control buffer (Ctrl) for 24 h (the experimental design is shown in Figure 1A). SiRNA against *Sphk1* significantly diminished *Sphk1* expression in both Ctrl- and AngII-treated groups as compared with siNC groups (Figure 1B). To assess whether AngII treatment induced hypertrophy of CMs in vitro, we examined the expression levels of the common pathological hypertrophy marker *natriuretic peptide type B* (*Nppb*) [18]. While AngII treatment significantly upregulated the expression of *Nppb*, it was markedly reduced by siSphk1 treatment in AngII-treated CMs (*p* < 0.05 for the interaction between AngII/Ctrl and siSphk1/siNC factors) (Figure 1B).

RNASeq analysis of AngII-treated CM transcriptomes revealed that *Sphk1* silencing significantly changed the expression levels of 2491 genes (false discovery rate (FDR) corrected *p*-value < 0.05), among which 1144 were upregulated and 1347 genes were downregulated (see Supplementary Material for summary statistics of all genes detected in the RNASeq analysis). Results concerning all 52 genes for which expression was >2.5 times up- or downregulated (FDR *p* < 0.05) by siRNA treatment are presented on a heatmap (Figure 1C).

### 2.2. Sphk1 Silencing Influences Pathways Related to CM Development and Proliferation

**Figure 2:**
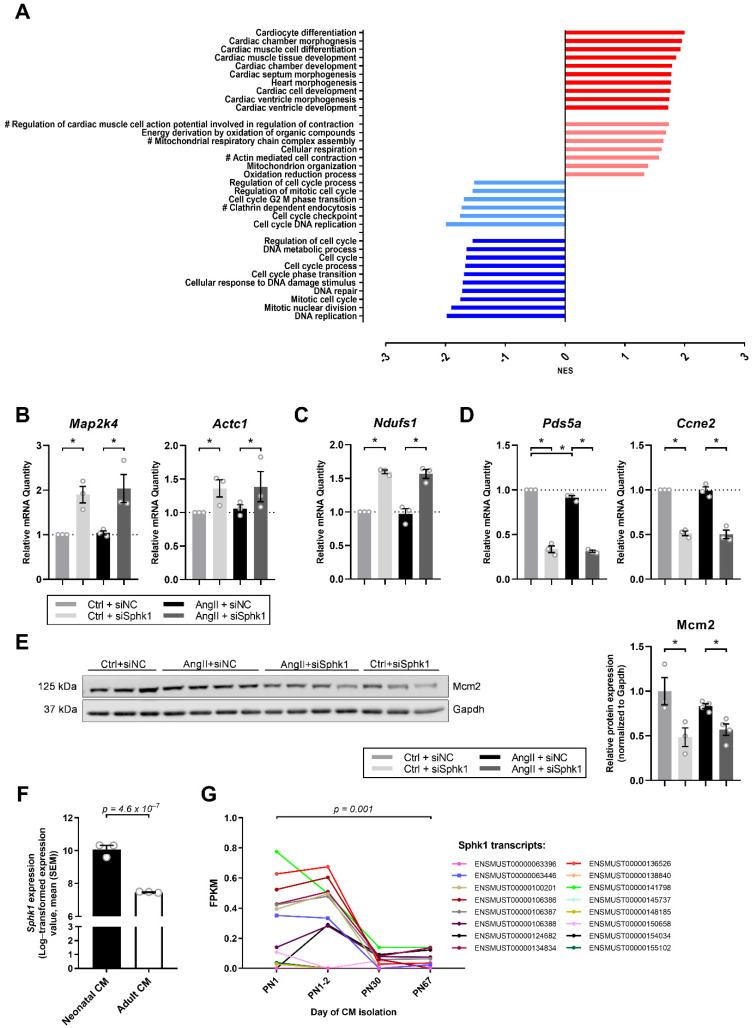
*Sphk1* silencing influences developmental and cell cycle-related pathways in neonatal CMs. (**A**) The first 10 most significantly (false discovery rate (FDR) *p* < 0.05) changed pathways with a normalized enrichment score (NES) of >1.5 (dark red) or <−1.5 (dark blue) from gene set enrichment analysis (GSEA) in AngII-treated (24 h) CMs transfected with siRNA against *Sphk1* (siSphk1) as compared with negative control siRNA (siNC); pathways related to CM maturation (selected on the basis of the study by Giudice [20]) up- (light red) or downregulated (light blue) at FDR *p* < 0.05 (except for the indicated (#) pathways, for which 0.05 < FDR *p* < 0.1) in AngII-treated (24 h) CMs transfected with siSphk1 as compared with siNC. (**B**) mRNA expression of genes related to cardiomyocyte development: *mitogen-activated protein kinase kinase 4* (*Map2k4*) and *actin alpha cardiac muscle 1* (*Actc1*), (**C**) mitochondrial function: *NADH:ubiquinone oxidoreductase core subunit S1* (*Ndufs1*) and (**D**) cardiomyocyte proliferation: *PDS5 cohesin associated factor A* (Pds5a) and *cyclin E2* (*Ccne2*) quantified using real-time polymerase chain reaction in neonatal CMs transfected with siNC or siSphk1 and treated with AngII or a control buffer (Ctrl) for 24 h. Each point corresponds to an independent experiment (biological replicates). (**E**) Western blot and densitometric analysis of the expression of Mini-Chromosome Maintenance Protein 2 (Mcm2) in neonatal CMs transfected with siNC or siSphk1 and treated with AngII or a control buffer (Ctrl) for 24 h. Protein expression was normalized to GAPDH; (**F**) *Sphk1* expression in neonatal and adult mouse CMs according to Cattaneo et al. [21]. Each point corresponds to an independent experiment. (**G**) Expression, measured as fragments per kilobase of exon model per million reads mapped (FPKM), of various *Sphk1* transcripts in murine CMs at four different postnatal time points (postnatal (PN) Day 1, PN1-2, PN30 and PN67) according to Giudice et al. [20]. All data are represented as means ± SEM. * *p* < 0.05.

Subsequent Gene Set Enrichment analysis, using RNASeq data, revealed that *Sphk1* silencing primarily influences pathways related to cardiac development and proliferation. Among the first 10 most significantly changed pathways with normalized enrichment score (NES) of >1.5, all were associated with cardiac morphogenesis, differentiation or development, while among the first 10 most significantly changed pathways with a NES of <−1.5, all were related to cell cycle regulation (Figure 2A). Moreover, on the basis of the study by Giudice et al., which analyzed global transcriptomic changes and pathways altered during murine CM maturation [20], we identified those that were changed in the same direction in our experimental model. Analysis revealed that *Sphk1* silencing may have an impact on CM contractility, metabolism and mitochondrial function and may influence endocytosis in CMs (Figure 2A).

Real-time polymerase chain reaction experiments confirmed that *Sphk1* silencing in AngII-treated neonatal CMs induces mRNA expression of genes from cardiac development pathways such as *mitogen-activated protein kinase kinase 4* (*Map2k4*) and *actin alpha cardiac muscle 1* (*Actc1*) (Figure 2B), as well as from pathways related to mitochondrial activity and metabolism e.g., *NADH:ubiquinone oxidoreductase core subunit S1* (*Ndufs1*) (Figure 2C), while it reduces mRNA expression of genes from cell cycle-related pathways such as *PDS5 cohesin associated factor A* (*Pds5a*) and *cyclin E2* (*Ccne2*) (Figure 2D). Importantly, similar significant effects of *Sphk1* silencing were observed in the Ctrl group not treated with AngII (Figure 2B–D). What is more, AngII-treatment triggered a slight but significant downregulation of *Pds5a* as compared with the Ctrl (Figure 2D).

To examine the effect of *Sphk1* silencing on neonatal CM proliferation, we tested the expression of the commonly used proliferation marker Mini-Chromosome Maintenance Protein 2 (Mcm2), which was downregulated in siSphk1-transfected CMs as compared with siNC-transfected CMs in both AngII and Ctrl conditions (Figure 2E). This is in agreement with the study of Giudice et al., which found a time-dependent decline in *Mcm2* transcript expression in developing CMs postnatally [20].

The above results demonstrated that *Sphk1* may be important for neonatal cardiomyocyte differentiation and maturation early after birth, irrespective of the presence of AngII. To test such a hypothesis, we analyzed studies which compared the global transcriptomic profiles of neonatal versus adult CMs. Interestingly, data from the study of Cattaneo et al. showed a significantly reduced level of *Sphk1* in adult murine CMs as compared with neonatal murine CMs (Figure 2F) [21]. Moreover, data from the study of Giudice et al. demonstrated a time-dependent decline in the expression of 16 different *Sphk1* transcripts using four different postnatal time points (Figure 2G) [20].

### 2.3. Key Signaling Pathways Mediate the Effects of Sphk1 Silencing in Neonatal CMs

**Figure 3:**
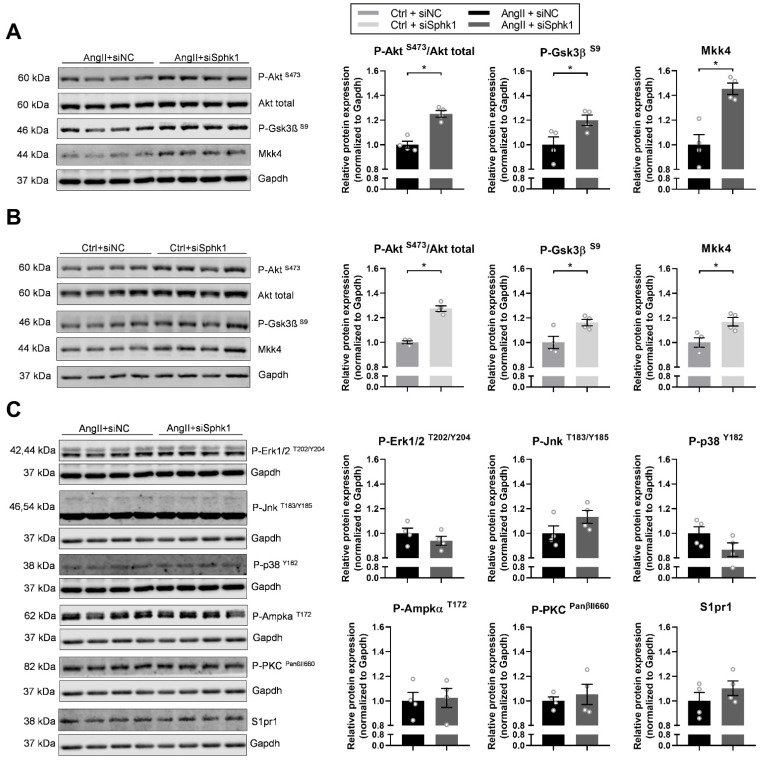
Key signaling pathways mediating the effects of *Sphk1* silencing in neonatal CMs. (**A**) Western blot and densitometric analysis of the expression of P-Akt^S473^, phosphorylated glycogen synthase kinase 3 beta (P-Gsk3β^S9^) and mitogen-activated protein kinase (MAPK) kinase 4 (Mkk4) in siSphk1- or siNC-transfected and AngII- or (**B**) Ctrl-treated (24 h) neonatal CMs. Protein expression was normalized to GAPDH. (**C**) Western blot and densitometric analysis of the expression of phosphorylated extracellular signal-regulated kinases 1/2 (P-Erk1/2^T202/Y204^), phosphorylated c-Jun *N*-terminal kinases (P-Jnk^T183/Y185^), phosphorylated p38 mitogen-activated protein kinase (P-p38^Y182^), phosphorylated 5′AMP-activated protein kinase alpha (P-Ampkα^T172^), phosphorylated protein kinase C (P-PKC^PanβII660^) and S1P receptor type 1 (S1pr1) in siSphk1- or siNC-transfected and AngII-treated (24 h) neonatal CMs. Protein expression was normalized to GAPDH. All data are represented as means ± SEM. * *p* < 0.05.

To investigate Sphk1-mediated signaling in neonatal CMs, we sought to examine the activation status of signaling pathways that are important for cardiomyocyte maturation. *Sphk1* silencing significantly upregulated Akt-mediated signaling in both AngII- (Figure 3A) and Ctrl-treated CMs (Figure 3B), as evidenced by an increase in Akt phosphorylation at Serine 473. Accordingly, we observed a significant upregulation in the phosphorylation of glycogen synthase kinase 3 beta at Serine 9 (P-Gsk3β^S9^) after *Sphk1* silencing in AngII-treated CMs (Figure 3A), as well as in Ctrl-treated CMs (Figure 3B), which suggests reduced Gsk3β activity.

Interestingly, in line with the observed upregulation of *Map2k4* mRNA expression after *Sphk1* silencing, there was a significant increase in the expression of its protein product—dual specificity mitogen-activated protein kinase (MAPK) kinase 4 (Mkk4)—in AngII-treated (Figure 3A), as well as in Ctrl CMs (Figure 3B), which may suggest a novel role of MAPK signaling in *Sphk1*-mediated effects in neonatal CMs.

Additionally, to further test MAP kinase signaling cascade, the expression of phosphorylated forms of extracellular signal-regulated kinases 1/2 (Erk1/2), c-Jun *N*-terminal kinases (Jnks) and p38 mitogen-activated protein kinase (p38) was examined. However, we found no significant changes after *Sphk1* silencing in P-Erk1/2, P-Jnk and P-p38 expression levels either in AngII-treated (Figure 3C) or in Ctrl CMs (data not shown). Similarly, *Sphk1* silencing had no effect on the expression of phosphorylated protein kinase C (P-PKC^PanβII660^) and phosphorylated 5′AMP-activated protein kinase alpha (P-Ampkα^T172^) either in AngII-treated (Figure 3C) or in Ctrl CMs (data not shown).

Furthermore, we tested whether *Sphk1* silencing has an impact on the expression of S1P receptor type 1 (S1pr1), the most abundantly expressed S1P receptor in CMs [22], and we found no significant changes as examined by Western blotting either in AngII-treated CMs (Figure 3C) or in Ctrl CMs (data not shown).

## 3. Discussion

The present study revealed, for the first time, the potential involvement of *Sphk1* in the development of neonatal mouse CMs. Neonatal CMs lacking *Sphk1* displayed significantly altered transcriptomic and signaling profiles as compared with CMs transfected with control siRNA, which facilitated their postnatal maturation in both physiological and stress conditions.

Cardiac myocytes undergo dynamic changes during fetal and postnatal development, reaching a mature phenotype shortly after birth [23,24]. Neonatal mouse CMs exit the cell cycle within the first week of life and undergo the final stage of differentiation [25]. Cardiomyocyte maturation is characterized by myofibril maturation, changes in electrophysiology and Ca^2+^ handling, enhanced mitochondrial biogenesis and oxidative capacity, a shift from glucose to fatty acid metabolism and a proliferation-to-hypertrophy transition [23]. Numerous extracellular signaling molecules such as thyroid hormone (T3), vascular endothelial growth factor (Vegf) and insulin/insulin-like growth factor 1 (Igf1), as well as blood flow and oxygen and their downstream signaling, have been recognized as crucial regulators of physiological postnatal CM development [26]. However, to our knowledge, there are no available data suggesting the role of the Sphk1/S1P axis in neonatal CM maturation.

Our results demonstrate that *Sphk1* silencing may favor the neonatal to adult CM transition. Transcriptomic and subsequent pathway analysis of *Sphk1*-lacking AngII-treated CMs revealed a marked upregulation of cardiac developmental pathways, as well as downregulation of pathways related to cell proliferation, which is characteristic of maturing CMs [27]. Additionally, *Sphk1* silencing altered pathways related to CM contractility, metabolism/mitochondrial function and endocytosis in a similar direction as that observed in maturing murine CMs postnatally in the study by Giudice et al. [20]. Among the leading edge genes, the most strongly contributing to the observed enrichment signal of the cardiac development-related pathways were *Map2k4* and *Actc1*. The latter encodes Alpha-cardiac actin, an alpha-actin isoform predominantly expressed in adult CMs [28], whereas *Map2k4* encoding Mkk4 (also known as Mek4 or Sek1), a kinase involved in the MAPK signaling cascade, is required for cardiomyocyte differentiation from embryonic stem cells [29]. What is more, Liu et al. have shown that Mkk4 prevents the transition from adaptive to maladaptive cardiac hypertrophy; however, it had no effect on physiological hypertrophy development [30]. Moreover, *Sphk1* silencing markedly induced the expression of *Ndufs1* (a leading edge gene in pathways related to mitochondrial activity), which encodes one of the subunits of NADH:ubiquinone oxidoreductase, an enzyme engaged in the mitochondrial oxidative metabolism, which is the main source of adenosine triphosphate (ATP) in high-energy-demanding, terminally differentiated CMs [31]. Cell cycle-related genes such *Pds5a* and *Ccne2*, encoding *Sister chromatid cohesion protein PDS5 homolog A* and *G1/S-specific cyclin-E2*, respectively, have well-known roles in cell division [24,32]. Their downregulation in *Sphk1*-lacking CMs indicates the diminished proliferation potential of such cells. This was further confirmed by the protein expression analysis of Mcm2, which is a widely acknowledged proliferation marker, playing a major role in the initiation of DNA replication [33]. The function of Sphk1 as a regulator of cell division is well documented [34,35], but not in the context of early postnatal CM maturation. Importantly, all cardiac development and mitochondrial activity-related genes, as well as proliferation-related genes and protein markers were similarly altered in both control and stress (i.e., AngII-induced) conditions, suggesting that AngII does not interact with Sphk1 during maturation of neonatal CMs.

Interestingly, a literature review and analysis of the available data from studies comparing neonatal and adult CM transcriptomes [20,21] allowed us to reveal a time-dependent decline in *Sphk1* expression, parallel to the growing degree of CM differentiation status. This may additionally support an important role of *Sphk1* in postnatal CM development, and it would be of high interest to test whether and how observed differences correspond to the level and activity of Sphk1 protein postnatally.

Postnatal cardiomyocyte development is orchestrated by a complex net of signaling pathways [23,24,26]. However, extracellular signaling initiated by thyroid hormone, Vegf or insulin/Igf1 all merge with downstream Akt signaling, making this serine–threonine kinase a central regulator of CM maturation. Indeed, numerous studies have shown that Akt positively regulates physiological heart growth [36,37]. Cardiomyocyte hypertrophy is also orchestrated by MAP signaling kinases, with Erk1/2 activation being assigned to both physiological and pathological types of hypertrophy [38,39,40], and Jnk and p38 kinases being characteristic for stress-induced pathological hypertrophy [26].

Importantly, our study revealed that the lack of *Sphk1* significantly upregulated Akt signaling in neonatal CMs and had no effect on Erk1/2 activation. PI3K/Akt signaling branches into one path leading to the mammalian target of rapamycin (mTOR) and another leading to Glycogen synthase kinase-3β (Gsk3β), both of which control gene transcription and protein synthesis in CMs and thus regulate CM maturation [26,41]. To induce growth-promoting signaling in CMs, mTOR signaling is activated, while Gsk3β becomes inhibited by Akt. A study by Michael et al. revealed that overexpression of Gsk3β in cardiac muscle leads to a small heart phenotype and reduces cardiac contractility due to impaired calcium handling, identifying Gsk3β as a critical negative regulator of physiological cardiomyocyte growth [42]. What is more, cardiac-specific inactivation of *Gsk3β* results in physiological hypertrophy of the heart [43]. Our study shows that Akt upregulation is accompanied by concurrent inhibition of Gsk3β, reflected by the increase in the level of inhibitory phosphorylation, which further supports the hypothesis that a lack of *Sphk1* may accelerate the neonatal to adult CM transition.

Interestingly, we observed an alteration in the arm of MAPK signaling involving Mkk4. Canonical Mkk4 downstream targets are Jnks, as well as p38 kinases, which are activated in pathological hypertrophy [2,44]. In our study, despite the markedly induced expression of Mkk4 in *Sphk1*-lacking CMs, no change either in P-Jnk or in P-p38 kinase expression was observed, which indicates that Mkk4 may regulate distinct downstream signaling events in our experimental model. Interestingly, a study by Shao et al. reported that in rodent CMs expressing constitutively active Mkk4, there was a strong upregulation of Akt signaling [45]. This observation suggests that Mkk4 may potentially, directly or indirectly, target Akt in neonatal CMs. Of note, the lack of *Sphk1* in neonatal CMs did not alter the activation status of other important maturation kinases, such as AMP-activated protein kinase (Ampkα), regulating energy metabolism [46,47], and PKC, controlling cardiac contractile function [48,49,50].

Surprisingly, all the observed effects of *Sphk1* silencing in neonatal CMs were similar in control and stress (AngII-induced) conditions. This suggests that *Sphk1* silencing accelerates CM maturation regardless of AngII’s presence. Interestingly, besides the commonly acknowledged function of AngII as a pathological hypertrophy inducer, AngII signaling is involved in physiological hypertrophy of embryonic as well as neonatal CMs [16,51,52]. Importantly, Diniz et al. reported that thyroid hormone (T3)-dependent physiological hypertrophy is mediated by an increase in the expression of AngI/II and angiotensin type I receptor (Agtr1) in CMs, and blocking of Agtr1 prevents T3-induced Akt/Gsk3β/mTOR pro-growth signaling [16]. Taken together, besides known differences in their molecular mechanisms, it may be challenging to distinguish the physiological and pathological types of hypertrophy in cultured neonatal CMs, as they might run in parallel. Interestingly, studies by Kemi et al. revealed that the activation or inactivation of cardiac Akt/mTOR signaling may serve as a tool to differentiate between physiological and pathological hypertrophy [53]. Since our previous studies clearly showed that both genetic and pharmacological inhibition of *Sphk1* prevented the development of hypertension-induced left ventricular cardiac hypertrophy, the primary aim of our study was to investigate the molecular effects of *Sphk1* silencing in AngII-induced hypertrophy in vitro; however the usage of a neonatal CMs model allowed us to additionally recognize a heretofore unknown role of *Sphk1* in neonatal CM development. Additionally, we have proposed a novel mechanism, which, besides conventional Akt signaling, involves *Map2k4*/Mkk4 as a positive regulator of CM maturation.

In summary, the current study found that silencing of *Sphk1* may induce various developmental pathways in neonatal CMs in vitro. It is of future interest to investigate the exact mechanism by which *Sphk1* silencing influences CM development; i.e., whether it modulates receptor-dependent S1P signaling or intracellular S1P action. Additionally, the long-term consequences of *Sphk1* silencing in the neonatal heart need to be investigated in vivo, because the altered maturation of CMs may influence cardiac functioning in adulthood [54]. What is more, cardiomyocyte maturation studies are gaining growing interest nowadays, considering its relevance in cardiac regenerative medicine [23]. Current technology provides effective protocols for the differentiation of CMs from induced pluripotent stem cells (iPSCs); however, such cells display an immature phenotype, which restricts their usage in cardiac regeneration studies [55]. This makes studies considering *Sphk1* in CM maturation even more interesting for further investigation.

## 4. Materials and Methods

### 4.1. Neonatal Mouse Cardiomyocytes

Neonatal cardiomyocytes (CMs) were isolated from cardiac ventricles of 1–3-day-old C57BL6/J mice by using the Pierce Primary Cardiomyocyte Isolation Kit (#88281, ThermoFisher, Waltham, MA, USA) according to the manufacturer’s protocol. Briefly, cells were cultured on 48-well plates at a seeding density of 400,000 cells/well in DMEM for primary cell isolation containing 10% FBS and penicillin (100 U/mL)/streptomycin (100 U/mL) (complete medium) using standardized parameters: 37 °C, 5% CO_2_ and 95% humidity [56,57]. After 24 h, the medium was replaced with fresh complete medium additionally containing a cardiomyocyte growth supplement (CGS). After 24 h, cells were washed with PBS, fresh complete medium containing CGS was added, and cells were transfected with 3 pmol/well of Silencer Select siRNA against murine Sphk1 (siSphk1) or silencer select negative control (siNC) (ThermoFisher, Waltham, MA, USA) using Lipofectamine RNAiMAX transfection reagent (ThermoFisher, Waltham, MA, USA) according to the manufacturer protocol. After 24 h, cells were treated with AngII (1 µM) or a control buffer (saline) for the next 24 h. Subsequently, cells were washed with PBS and lysed with 300 μL of TriReagent Solution (ThermoFisher, Waltham, MA, USA). Cell lysates were stored at −80 °C for subsequent RNA and protein isolation. Three independent CM isolations (biological replicates) and subsequent treatments described above were performed (in quadruplicate for each treatment group for each isolation).

### 4.2. RNASeq Analysis

Total RNA from CMs transfected with siSphk1 or siNC and treated with a control buffer (Ctrl) or AngII was isolated using Direct-zol RNA Miniprep kit and treated with DNase I (Zymo Research, Irvine, CA, USA). mRNA was isolated using NEBNext Poly(A) mRNA Magnetic Isolation Module (New England Biolabs, Ipswich, MA, USA). The mRNA library was prepared using the NEBNextUltra II Directional RNA Library Prep Kit for Illumina. Sequencing was performed on HiSeq4000 (Illumina, San Diego, CA, USA) with 150 bp paired-end reads (PE150). Bioinformatic analysis included trimming of adapters, polyA and polyT tails using Cutadapt [58]. RNASeq reads were then mapped to genes according to the reference genome (*Mus musculus* M25, GRCm38.p6 downloaded from GENCODE) using Hisat2 software [59]. Reads were counted with HTseq [60].

### 4.3. Real Time PCR

Total RNA from CMs transfected with siSphk1 or siNC and treated with AngII or a control buffer (Ctrl) was isolated using a Direct-zol RNA Miniprep kit and treated with DNase I (Zymo Research, Irvine, CA, USA). Reverse transcription was performed using the High-Capacity cDNA Reverse Transcription Kit (Applied Biosystems, Foster City, CA, USA). Real-time PCR reactions were performed using commercially available TaqMan assays, Taqman Gene Expression Master Mix and the 7900HT instrument (Applied Biosystems, Foster City, CA, USA). TATA box binding protein (Tbp) was used as a housekeeping gene for all performed real-time PCR reactions. Ct values were calculated using RQ Manager (Applied Biosystems, Foster City, CA, USA).

### 4.4. Analyses of Murine Sphk1 Expression in CMs over Time

We used 2 published datasets from the GEO database [61], which analyzed, among others, Sphk1 mRNA expression using either microarray (Series GSE44829, [21]) or RNASeq (Series GSE49906, [20]) approaches. Cattaneo and colleagues analyzed transcriptomes from neonatal (postnatal (PN) Day 1) CMs as well as adult (2 months old) murine CMs using the Illumina microarray approach [21]. Giudice and colleagues analyzed the expression of mRNA transcripts, including various splicing isoforms, in murine CMs isolated at PN (postnatal day) 1, PN1-2, PN30 and PN67 days using the RNASeq approach [20].

### 4.5. Western Blotting

Protein was isolated from cells lysed in TRI Reagent Solution according to the manufacturer’s protocol and suspended in a buffer containing EDTA (20 mM), NaCl (140 mM), Tris (100 mM), 5% sodium dodecyl sulfate (SDS) and Halt Protease and the Phosphatase Inhibitor Cocktail (ThermoFisher, Waltham, MA, USA) [62]. Protein concentration was measured using a Pierce BCA Protein Assay Kit (ThermoFisher, Waltham, MA, USA). Electrophoretic separation was performed using 15–25 μg of protein loaded on Bolt 4–12% Bis-Tris Plus gels (ThermoFisher, Waltham, MA, USA). Subsequently, protein was transferred to a 0.45 μm nitrocellulose membrane, which was then horizontally cut into strips in order to enable detection of several proteins using a single membrane. Nonspecific binding sites were blocked by incubation with 5% low-fat milk in a Tris-buffered saline (TBS) solution for 1 h at room temperature (RT) and washed with TBS containing Tween20 (0.01%). Membranes were then incubated overnight (4 °C) with the primary antibodies listed below. After washing, membranes were incubated with the corresponding IRDye 800CW/680RD secondary antibodies (LI-COR Biosciences, Lincoln, NE, USA) for 1 h at RT. Fluorescence signals were visualized with the Odyssey Fc reader (LI-COR Biosciences, Lincoln, NE, USA). Densitometric analysis was performed using Image Studio (ver. 5.2) software. GAPDH was used as a stable housekeeping protein not affected by either AngII or siRNA treatment in neonatal CMs.

Anti-Mcm2 (#3619, dilution 1:1000), Anti-Mkk4 (#9152, 1:1000), Anti-P-Akt^S473^ (#4060, 1:1000), Anti-Akt total (#9272, 1:1000), Anti-P-Gsk3β^S9^ (#9323, 1:1000), Anti-P-Erk1/2^Thr202/Tyr204^ (#4370, 1:2000), Anti-P-Ampkα^T172^ (#2535, 1:1000) and Anti-P-PKC^PanβIISer660^ (#9371, 1:750) antibodies were purchased from Cell Signaling Technology (Danvers, MA, USA). Anti-P-Jnk^T183/Y185^ (sc-6254, 1:500) and Anti-P-p38^Y182^ (sc-7973, 1:500) antibodies was purchased from Santa Cruz Biotechnology (Dallas, TX, USA). Anti-S1pr1 (#PA1-1040, 1:300) and Anti-GAPDH (#MA5-15738, 1:4000) antibodies were purchased from ThermoFisher (Waltham, MA, USA).

### 4.6. Statistical Analysis

Differential gene expression analysis from the RNASeq experiment was performed using the DESeq2 package in R (version 3.5.1) [63]. Gene set enrichment analysis (GSEA) included all genes with complete summary statistics following DESeq2 analysis (see the Supplementary Material for complete results from DESeq2 and GSEA) and was performed using the fgsea package in R using gene sets derived from the GO Biological Process ontology [64,65,66]. A linear mixed-effect model was used for analysis of PCR data, including the interaction between two factors, i.e., siRNA and AngII treatments. A *t*-test was used for analysis of protein expression data. All statistical tests were performed using IBM SPSS Statistics (version 25) or GraphPad Prism (version 8). Results are presented as means ± SEM (*p* values < 0.05 were considered statistically significant).

## Data Availability

Raw unprocessed gene counts, as well as all the results from RNASeq and Gene Set Enrichment analyses, are available in the Supplementary Material.

## Supplementary Materials

Supplementary Materials can be found at https://www.mdpi.com/article/10.3390/ijms22073616/s1.
